# Objective and personalized longitudinal assessment of a pregnant patient with post severe brain trauma

**DOI:** 10.3389/fnhum.2015.00128

**Published:** 2015-03-17

**Authors:** Elizabeth B. Torres, Brian Lande

**Affiliations:** ^1^Department of Psychology, Computational Biomedicine Imaging and Modeling Center, Computer Science, Neuroscience and Rutgers Center for Cognitive Science, Rutgers the State University of New JerseyNew Brunswick, NJ, USA; ^2^Department of Computer Science, University of CaliforniaSanta Cruz, CA, USA

**Keywords:** coma, pregnancy, brain trauma, wearable sensors, analytics, statistics

## Abstract

**Background**: Following severe trauma to the brain (whether internally generated by seizures, tumors or externally caused by collision with or penetration of objects) individuals may experience initial coma state followed by slow recovery and rehabilitation treatment. At present there is no objective biometric to track the daily progression of the person for extended periods of time.

**Objective**: We introduce new analytical techniques to process data from physically wearable sensors and help track the longitudinal progression of motions and physiological states upon the brain trauma.

**Setting and Participant**: The data used to illustrate the methods were collected at the hospital settings from a pregnant patient in coma state. The patient had brain trauma from a large debilitating seizure due to a large tumor in the right pre-frontal lobe.

**Main Measures**: We registered the wrist motions and the surface-skin-temperature across several daily sessions in four consecutive months. A new statistical technique is introduced for personalized analyses of the rates of change of the stochastic signatures of these patterns.

**Results**: We detected asymmetries in the wrists’ data that identified in the dominant limb critical points of change in physiological and motor control states. These patterns could blindly identify the time preceding the baby’s delivery by C-section when the patient systematically brought her hand to her abdominal area. Changes in temperature were sharp and accompanied by systematic changes in the statistics of the motions that rendered her dominant wrist’s micro-movements more systematically reliable and predictable than those of the non-dominant writst.

**Conclusions**: The new analytics paired with wearable sensing technology may help track the day-by-day individual progression of a patient with post brain trauma in clinical settings and in the home environment.

## Introduction

According to the Centers for Disease Control (CDC) severe trauma to the brain is a contributing factor to a third (30%) of all injury-related deaths in the US (Carroll et al., 2012; Centers for Disease Control and Prevention (CDC), 2013), thus posing a large societal and economic toll (Finkelstein et al., 2006). Non-fatal brain trauma may result in immediate unconsciousness (coma) and amnesia states followed by slow recovery with subsequent extended periods of impairments in one or more general functional areas. Trauma may be due to internal insult caused by seizures, tumors, among other factors. Trauma may also be due to collision with an external object (static or in motion). Different prognosis and subsequent states emerge from different types of injuries to the brain. These may include impaired cognitive and/or motor functions as well as impaired sensations and/or emotional responses. Physicians and researchers now generally recognize that the spectrum of disorders related to coma can be more broadly defined as a range of disorders of consciousness (DOC) that can be mapped onto a multi-dimensional space primarily defined by cognitive and motor impairments.

In many cases the initial coma state may evolve towards improved levels of consciousness and physical function such as a minimally conscious state (MCS). To assess coma and impaired consciousness in the early stages of post brain trauma there are several clinical tools based on reports from observation. These include the Glasgow Coma Scale (GCS), Coma Recovery Scale—Revised (CRS-R), the Abbreviated Injury Scale (AIS) and the Trauma Score or Abbreviated Trauma Score, among others. These observational tools can also be used to track progress while at the hospital or during subsequent visits, in cases where the patient improves and undergoes rehabilitation at home. Other tools used in the hospital settings include objective assessments of the brain condition using imaging techniques. The use of these techniques is however limited to a few times per year, due primarily to their cost and regional availability.

Upon recovery from the initial coma state, many patients undergo rehabilitation and eventually return home to be looked after by a caregiver and to continue receiving therapy. At that stage there are presently no objective tracking tools to help the caregivers, and/or the occupational and physical therapists assess the daily progression of the patient in response to treatments. The current assessments to track physical progress rely primarily on observation (e.g., the use of inventories such as the Western Neuro Sensory Stimulation Profile, WNSSP among others). Yet the human eye has limited capacity to detect subtle changes in physical motions that could signal improvement, or call for immediate attention to some sharp change in physiological states. For example, physicians and therapists look for eye opening to detect changes in arousal and behavioral command following and/or changes in spontaneous/reflexive movement to detect changes in awareness. Diagnoses of changes in states of awareness or arousal based on clinical observation alone have high rates of diagnostic error, approximately 40% (Schnakers et al., 2009). There is no way to objectively track the longitudinal rate of change of the person’s patterns so critical information is being missed that could help the patient and caregivers cope. In particular health insurance companies require evidence-based improvements for coverage of therapies but under the present observational methods it is a challenge to report accurate and reproducible results.

With the advent of wearable sensing technology it may be possible to use motion tracking in combination with other physiologically relevant signals (temperature, electrodermal activity, heart beat variability, etc.) to help medical personnel and care givers assess the patient’s mental and physical states daily, as they fluctuate, both during the hospitalization period and after discharge, when the patient goes into rehabilitation.

Wearable sensors are now ubiquitous in our lives. They are present in our smart phones, smart tablets, wellness and fitness bracelets, etc. Yet the current analytical techniques embedded in such devices have been recently called into question as somewhat inaccurate and occasionally misleading. Such methods may be acceptable to track fitness and wellness, but they may lack the reliability necessary to be adopted as standard metrics in the clinical domain. New analytical techniques to be embedded in wearable sensors are needed to help caregivers and medical personnel track the evolution of patients with severe post trauma to the brain. In this paper we introduce new personalized statistical methods that may be of help in tracking the progression of patients with brain trauma (independent of the type of trauma involved). We illustrate the methods with data from a pregnant patient who underwent severe brain trauma, slipped into a coma and had her baby successfully delivered by C-section.

## Methods

All methods and measurements presented in this study have been approved by the Rutgers IRB Committee in accordance with the Helsinki Act.

## Patient

### Timeline of the Patient as Reported by Her Doctors

**Figure 1:**
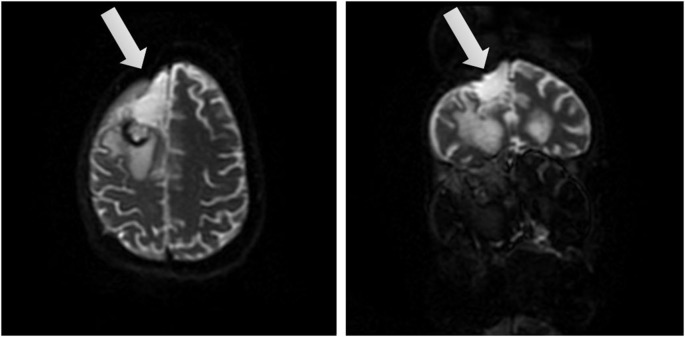
**Brain views after tumor removal**. Two different views of the right frontal lobe upon removal of the tumor (affected area marked by arrow).

AB is a 39-year-old, right handed woman who was pregnant when diagnosed with a grade 2 oligoastrocytoma on 03/05/14 after worsening headaches, fatigue, nausea and some degree of confusion which prompted an MRI scan. The MRI revealed on 03/07/14 a right frontal lobe mass lesion (8.5 × 5) with characteristics suggestive of oligodendroglioma. Surgical excision was recommended by the neurologist and scheduled for 03/12/14 in consultation with her high-risk Ob/Gyn. On the morning of 03/13/14 AB suffered an acute neurological decompensation with loss of consciousness and nonreactive dilated right pupil, sluggishly responsive pupil and decorticate posturing. It was thought that she had a seizure. She was intubated and given Mannitol and hyperventilated for probable increased intracranial pressure. A STAT CT of the brain revealed cerebral edema with uncal herniation. She underwent surgical decompression, a right hemicraniectomy with tumor debulking (see Figure 1 showing brain after surgical removal of the tumor). EEG upon surgery did not reveal seizure activity. Postoperative exams included decerebrate posturing, CT and MRI. These tests revealed extensive hemorrhagic infarct or cytotoxic edema involving multiple vascular territories in the bilateral parietal temporal and occipital lobes, as well as bilateral (right more than left) thalami. Small foci of ischemia were also found on the right mid-brain, pons and right cerebellar hemisphere.

During the first week post-operation, AB remained unconscious. Her GCS was 3. Even under comatose state she displayed spontaneous eye opening and movements in the extremities. Some examiners reported hand movement on command on 03/20/14, but subsequent reports have been inconclusive, possibly due to delays in response and inconsistencies in responses. On 03/20/14 an external ventricular drain was camped. An MRI on 03/23/14 revealed interval development of a large psudomeningocele at the right hemicraniectomy site. The external ventricular drain was removed on 03/24/14.

On 05/22/14 AB underwent a C-section delivery of a healthy baby boy. On 05/28/14 a percutaneous endoscopic jejunostomy tube was placed. She was transferred from the hospital to a rehabilitation hospital for neurorehabilitation.

At the hospital she had fever on 06/25/14 due to an infection. She underwent a course of antibiotics. A clot in her IVC was revealed by ultrasound on 06/26/14. She was fully anticoagulated prophylactically and fully anticoagulated with Lovenox.

Patient AB is on a trach collar. Her ABG on 06/30/14 showed adequate oxygenation. Her weekly scores on the WNSSP from June 4th 2014 till October 8th 2014 are reported on Table 1. The discharge medications are reported below.

**Table 1:** **Weekly scores from the Western Neuro Sensory Stimulation Profile (WNSSP) commonly used to track changes in neural sensory processing**.

Month	(Day) WNSSP
June	(4) 11	(11) 10	(18) 26	(25) 27	
July	(2) 27	(10) 22	(17) 22	(24) 22	(31) 29
August	(6) 13	(13) 14	(20) 5	(27) 17	
September	(3) 7	(10) 3	(17) 10	(24) 3	
October	(1) 9	( 8) 14			

### Discharge Medications

*Medications administered per feeding tube*: Amantadine 150 mg, 50 mg in the AM and 100 mg noon; Desmopressin 0.1 mg per day; Docusate 2 mg per day; Ferrous sulfate 300 mg; Folic acid 1 mg; Glycopyrrolate 0.5 mg; Keppra 1000 mg; Multivitamin (1 tablet); Potassium chloride 20 mEq; Senna two tabs; Vitamin D3 2000 IU; Aquatears to both eyes four times a day; Chlorhexidine 15 ml for oral care 4 times daily; Meropenem 1 g IV q 8.

*Medications administered by subcutaneous bid*: Enoxaparin 50 mg and Vancomycin 1 g IV.

### Measurements

The wrist motions of patient AB were continuously captured in various daily sessions across the months of April till July 2014 using inertial measurement units IMU (APDM opal, Portland, OR). These IMU register linear and angular acceleration, a signal related to surface skin temperature, gyroscopic data, and magnetometer data at 128 Hz. The units are synchronized and operate through wireless technology in live streaming mode and also in robust logging mode. The former enables real time visualization of the synchronous data with no loss of data, while the latter allows the same without visualization of the recordings streamed in real time. We report data from the right and left wrists of the patient, synchronously recorded in robust logging mode (no data loss). Each session comprises several hours. Table 2 provides information on the number of hours per session when the data were registered. We describe statistical features of the data using new biometrics that connect acceleration-dependent motion and temperature-dependent data.

**Table 2:** **Number of hours recorded by the APDM sensors per each day session across the 4 months**.

	Day, hours		Day, hours		Day, hours		Day, hours		Day, hours		Day, hours		Day, hours	
**April**	24	7.09	25	7.22	26	7.16	29	6.41	
**May**	3	9.45	6	12.56	8	12.28	11	3.45	13	6.26	17	6.74	27	12.24
**June**	5	3.57	8	11.33	12	7.32	20	13.29	
**July**	1	9.34	9	7.06	12	9.43	15	4.32	17	5.37	19	7.14	

### Biometrics

**Figure 2:**
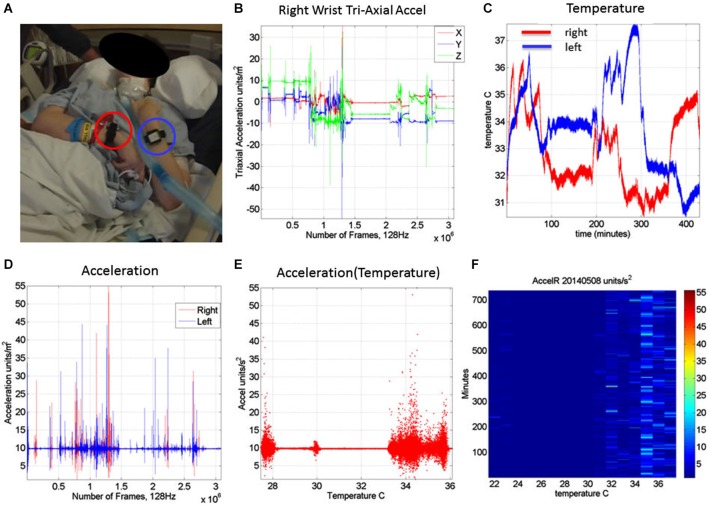
**Measurements: (A) Patient wearing the sensors in both wrists. (B)** Tri-axial acceleration measurements from one of the sensors during recording one session of 7.09 h. **(C)** Temperature measurements from both the left and right wrist sensors during the session. **(D)** Acceleration scalar obtained by computing the norm of each acceleration vector over time. **(E)** Acceleration plotted as a function of temperature (degree Celsius) for the full range of temperatures registered across the 7.09 h session of April 24th 2014. **(F)** Matrix of maximal deviations from the mean acceleration values registered on May 8th 2014 for the temperature range and time duration in minutes (for 12.28 h). For each minute and °C the motion content was registered. The color bar shows the range of the motion values (units/s^2^). The range of changes in temperature values was registered between 22°C and 38°C for that day’s recording session.

The motion patterns were analyzed along with those of the temperature values, both registered simultaneously by the sensors. We focus our analyses on the linear acceleration obtained from the tri-axial linear accelerometers. To this end we first express the linear acceleration as the time series of the norm of the three-dimensional vector of accelerations expressed as a function of the temperature range in each section. The patterns of variability of the maximal instantaneous deviations of the acceleration from the overall mean acceleration across the session were examined using distributional analyses previously described in other work involving velocity- and acceleration dependent signals (Torres, 2011, 2013a,b; Torres et al., 2013, 2014). Figure 2 shows representative data from the patient’s wrists. The location of the sensors is circled on the patient’s wrists in Figure 2A. Figure 2B shows the plots of the tri-axial acceleration profiles over several hours obtained on April 24th 2014 (see also Table 2). Figure 2C shows the profiles of temperature registered by the sensors while 1D shows the acceleration profiles. These are built as the time series of the instantaneous norm of the acceleration vector, $accel=\sqrt{{\left({\vec{a}}_{1}\right)}^{2}+{\left({\vec{a}}_{2}\right)}^{2}+{\left({\vec{a}}_{3}\right)}^{2}}$ in a given session. Here the *a_i_* are the tri-axial components along the *x, y, and z* axes.

**Figure 3:**
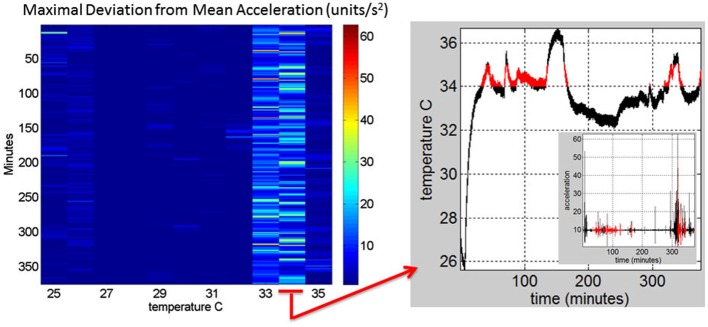
**Construction of the matrix containing the sensor data from a registered range of surface skin temperatures: Motion data from the tri-axial linear accelerometers are obtained continuously for each minute of recordings (128 Hz × 60 frames) in 6.26 h (375.6 min) for this session**. Each entry of the matrix contains the maximal deviation from the mean acceleration at each °C-interval (columns) and for each minute (rows) of the session. The right panel shows the 34–35°C-interval (red) and the inset shows the linear acceleration data corresponding to that temperature °C interval. On the left panel the 34–35°C-interval is marked to show the patterns of the maximal deviation from the mean acceleration over the session’s time length.

Figure 2E shows the scalar acceleration expressed as a function of the temperature range registered by the sensors. We take the mean acceleration value and the instantaneous maximal deviation from the overall mean of the session. These profiles are then obtained as a function of temperature. For each minute of the session all samples of the maximal deviation from the mean acceleration are obtained and plotted in matrix form in Figure 2F (shown for a session in May 8th 2014) for 12.28 h (739.6 min shown along the rows). The columns of the matrix show one-degree Celsius intervals spanning the range of temperatures for that session. Notice that the temperature output includes the skin surface temperature, the ambient temperature and the internal temperature of the sensors’ battery as it drains when motions are being registered. The color of each entry in the matrix reflects for each minute and degree interval the maximal amount of motion deviating from the mean acceleration (see color bar) in units/s^2^. Figure 3 illustrates the steps followed to build these matrices. The acceleration and temperature data is first harnessed in one-minute-long intervals (128 Hz × 60 s, 7,680 registered frames). For each degree the range of motion registered is obtained over time. The example in Figure 3 shows this for the 34–35°C-interval. All motion data occurring in that interval is harnessed (inset in right panel). Then for each minute and each °C the maximal deviation from the mean acceleration is obtained. Across the minutes and degrees, these are the entries of the matrix depicted in Figure 3. The color indicates the amount of motion maximally deviating from the mean acceleration of the session on May 8th.

**Figure 4:**
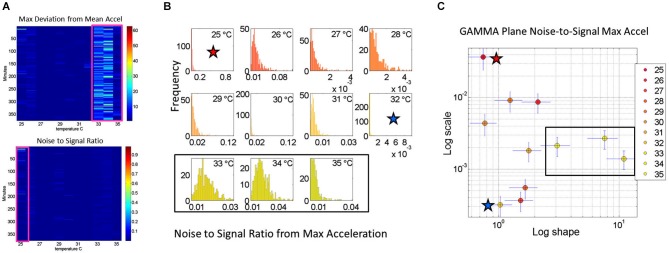
**Noise analyses to separate predictable and reliable from random and noisy motion data: The minute by minute variability is obtained for the maximal deviations from the mean acceleration, taken for each °C interval. (A)** Top panel is the matrix of maximal deviations from the mean linear acceleration (explained in Figure 2) within the temperature regime of motions. Bottom panel is the matrix of the noise-to-signal ratio (the Fano Factor: the estimated Gamma variance divided by the estimated Gamma mean) obtained from the estimated *shape* and *scale* parameters of the continuous Gamma family of probability distributions. The highest motion regime occurs between 33°C and 35°C. The highest noise regime occurs at 25°C while the lowest noise regime occurs at 32°C. **(B)** The frequency histograms of the noise-to-signal values are color coded in order of increasing temperature values. Colors are in correspondence to the points on the Gamma plane in **(C)**. The red star marks the highest noise-to-signal regime while the blue star marks the lowest regime. The temperature intervals containing the highest motion patterns are enclosed by a rectangle. These correspond to the three right most points in the Gamma plane (most systematic patterns), also enclosed within a rectangle.

In Figure 4 we continue to use the May 8th matrix to further illustrate the methods. We use the range from 33–35°C to show the statistics of the motion. For each °C we count the number of maximal deviations (peaks) across the session (6.26-h or 375 min along the rows of the matrix) and gather them in a frequency histogram. For each of the histograms representing the motions for each °C-interval we then fit a probability distribution function. Using maximum likelihood estimation (MLE) we obtain estimates of the shape (*a*) and the scale (*b*) parameters of the Gamma probability distribution with 95% confidence intervals. (We have used the continuous Gamma family of probability distributions in previous work to characterize the range of human motion variability across a range of neurological disorders and typical motions). From the Gamma estimated parameters we obtain the Gamma statistical parameters (mean and variance) and plot them on a (μ, σ)-plane. Each point represents the Gamma statistical parameters of the acceleration-dependent motions for a temperature °C-interval taken across the time length of the session.

### Noise-to-Signal Ratio Analyses

**Figure 5:**
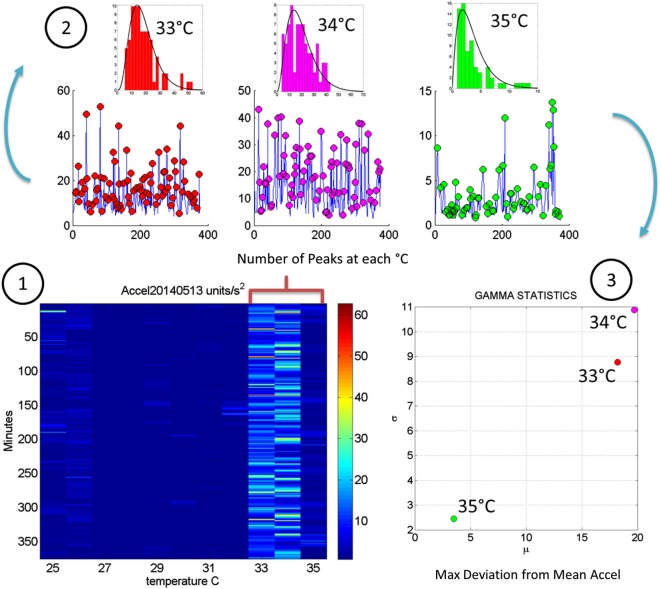
**Steps to analyze the patterns of acceleration variability:** (1) Build the matrix having in each entry the maximal deviations from the mean acceleration, as described in Figure 2, for each minute of the 6.26-h session and each °C-interval of the surface skin temperature range registered (2). For each column harness the peaks from the entries of the matrix with low and predictable noise-to-signal ratio. The frequency histogram of the peak deviations from the mean is obtained for each °C interval and the best fitting probability distribution function obtained (3). Obtain the empirically estimated Gamma mean and Gamma variance from the experimental data using the estimates of the shape and scale Gamma parameters (see text for details).

We also use for each minute (comprising the 60 s × 128 Hz frames per minute) the above mentioned approach to obtain for each entry in the matrix the Fano Factor. This is the variance divided by the mean, the noise to signal ratio. The resulting noise-to-signal ratio matrix corresponding to the motion matrix for the May 8th session is shown in Figure 5A bottom panel. Notice here that at 25°C the highest noise-to-signal level is revealed. Figure 5B shows the frequency histograms for each of the 11 columns of the matrix corresponding to each °C-interval. We mark the regimes with the highest (blue star) and lowest (red star) noise levels detected at 25°C and 32°C respectively. This immediately alerts us that not all motion from the accelerometers is physiologically relevant. At 25°C for example this session reveals a pattern of motion whereby the motion noise registered by these accelerometers overpowers the signal. The range from 33–35°C used in Figure 4 to illustrate the methods are also marked here to show their range of noise-to-signal. Using the MLE procedure we estimate the Gamma distribution shape and scale parameters of the distributions corresponding to the noise-to-signal values. This was done to determine the physiologically appropriate statistical regimes in the motion data to further analyze that data. These are regimes of temperature where the motion maintains minimal noise-to-signal ratios across the session, as opposed to the signal being overpowered by instrumentation noise. We plot the estimated shape and scale parameters on the Gamma plane with 95% confidence intervals in Figure 5C. The color code corresponds to the frequency histograms of Figure 5B and the legend reflects the corresponding temperature °C-interval for this May 8th session. The points corresponding to the shape value of 1 (log-log plot along the horizontal axis is 10°) are at the most random noise-to-signal levels. Those towards the right correspond to statistically more predictable (systematic) regimes of noise-to-signal levels (towards symmetric shapes of the distribution of the noise-to-signal ratios). Along the scale axis, higher values indicate higher levels of noise (highest marked by blue star in correspondence with the frequency histogram in Figure 5B). We also mark the 33–35°C temperature interval used in Figure 4B to illustrate the methods to isolate the physiologically relevant motion regimes and in correspondence with the frequency histograms of the noise-to-signal ratio in Figure 5B.

**Figure 6:**
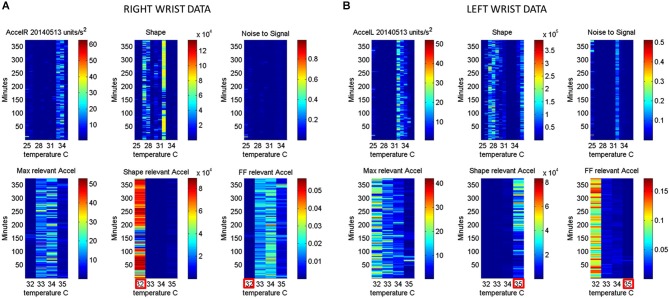
**Automatic Extraction of physiologically relevant data: (A)** Right wrist accelerometer and temperature data represented in matrix form and color coded by activity level in minute-by-minute intervals. As explained in Figure 2, in the first panel each entry of the matrix represents the maximal deviation from the mean acceleration for each given minute and °C. Color scale represents motion intensity from low (blue) to high (red). In the second panel each entry of the matrix represents the values of the shape parameter of the continuous Gamma family of probability distributions estimated from acceleration data. Each (i,j)-entry is the shape estimate for the i*^th^* minute the j*^th^* °C. Each entry in the third matrix, as explained in Figure 3, has the Fano Factor, the noise-to-signal ratio from the estimated Gamma mean and variance parameters for the i*^th^* minute and the j*^th^* °C. The lower panel contains the subset from the full range of surface skin temperature registered where the noise to signal is lower (minimal level enclosed by red square). In **A** (the right wrist) minimal noise in the acceleration is at 32°C (also the highest shape value, indicating most symmetric distribution of the motion parameter). In **(B)** (the left wrist) the noise-to-signal is low for 32–35°C, with 35°C having the lowest noise-to-signal regimes and the most systematic motions indicated by the higher values of the shape parameter.

In summary we first examine the motion statistical regimes for each minute and °C-interval (Figure 4) and then examine the noise-to-signal ratios corresponding to each of these acceleration-dependent motion entries (Figure 5). By combining temperature and motion of these sensors we automatically extract the range of physiologically relevant motion data to discriminate noise from signal. Then we can further perform our longitudinal activity tracking analyses to blindly detect relevant changes in skin surface temperature and to distinguish systematic from spontaneous acceleration-dependent motion patterns. Figure 6 summarizes in matrix form the separation between relevant data and noisy data using the May 8th session as an example.

### Automatic Blind Identification of Relevant Periods in the Longitudinal Data

**Figure 7:**
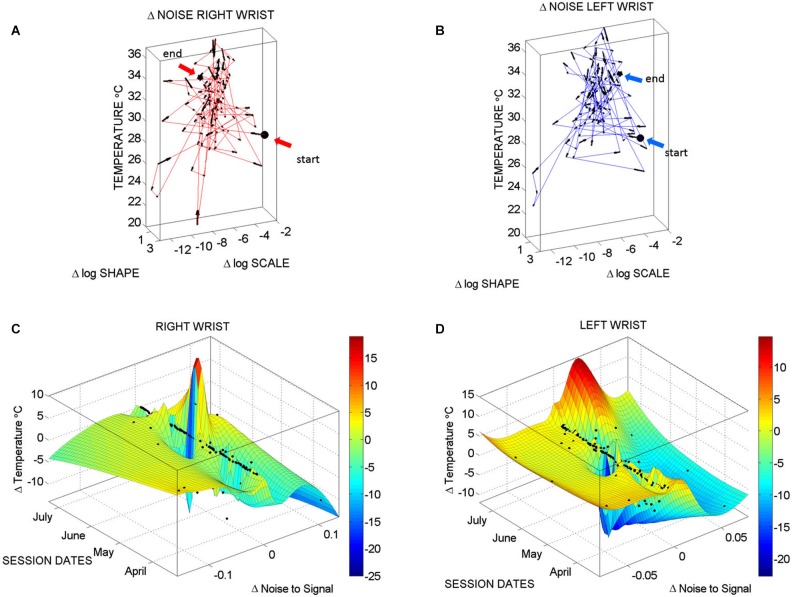
**Longitudinal changes in temperature and noise-to-signal ratio: (A) Stochastic trajectories of the right wrist variability in the acceleration data registered within the range of temperature with the lowest noise-to-signal ratio**. Arrows show the flow of directional change towards stable temperature regimes of low changes in the noise of the motion. This is marked by the size of the arrows from large at low temperature to small at higher temperature values (between 32–34°C). The starting and ending points of the stochastic trajectories are marked. **(B)** Same as in **(A)** for the left wrist. **(C)** Surface fit through 124 points from the motion data longitudinally obtained from the right wrist. These are the data with the lowest noise level in the maximal deviations from the mean acceleration. Notice that the area showing the sharpest rate of change in temperature and noise-to-signal levels was registered in May. **(D)** The surface fitting the 124 longitudinal data points from the left wrist shows decrease in the rate of change of temperature in May, followed by a gradually slow increase of this parameter across the subsequent months of June and July. In both cases there are also points aligned at near zero-change in noise-to-signal ratio for the motion. These are the points where the signatures of variability in the motion patterns registered by the sensors were more stable and had more steady state of temperature levels as well.

The analyses of the evolution in the patterns of noise-to-signal ratio for one session can be extended to each of the sessions to assess the *longitudinal* evolution of the physiologically relevant data in each session. Recall that these are the data combining the minimal noise-to-signal ratios across the various °C-intervals. Figure 7 depicts the longitudinal stochastic trajectory of the noise-to-signal ratios extracted from the motion data across all sessions. There are 124 measurements automatically extracted from 21 sessions registered across 4 months (spanning from April to July). In each session several temperature °C-intervals of low noise data were extracted and their shift in Gamma (*b)-scale* parameter levels obtained for each °C-interval along with their shift in the Gamma (*a*)-shape parameter corresponding to the frequency histogram of the motion’s noise-to-signal ratio and estimated using MLE. We point here that the Gamma (*b*)-scale parameter relates to the Fano Factor, the Gamma estimated variance divided by the Gamma estimated mean value. The former is *a.b* while the latter is *a.b*^2^. The Fano Factor is then *b*, which is the scale parameter. Thus we are examining the rate of change of the noise-to-signal ratio from the acceleration-dependent motions as they turn more or less random, and/or as they turn more or less systematic.

Figure 7A (right wrist) and Figure 7B (left wrist) show the 3-dimensional trajectories of the changes in these Gama parameters (X-Y log-log plane) along the temperature ranges (Z-axis °C) registered by the sensors. The vector field (black arrows) indicates the direction and the magnitude of the change in the reliability and predictability of the changes in the noise-to-signal ratio form the motion data. Low changes in values vs. high changes in values are better appreciated in Figures 7C,D along the surface fitted through the 124 points of physiologically relevant (low noise) data across all sessions. Along the Z-axis of these surfaces are the changes in temperature level. Notice that the right wrist had a dramatically sharp change in the month of May, while the left wrist had a gradual change in temperature from June onwards. We come back to this observation in the discussion section below. Points along the 0-change lines of temperature, scale and shape are steady states in each session.

### Identification of and Further Distributional Analyses in Critical Sessions

**Figure 8:**
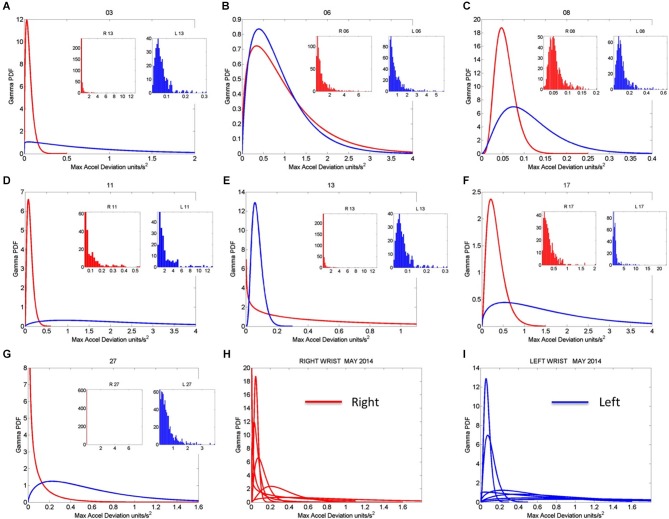
**Gamma pdf estimation from each session and temperature range having low noise-to-signal information in the month of May 2014. (A–G)** The estimation of the Gamma pdf’s across 7 sessions was done from the empirical data registered by the sensors at the wrist. Insets are the frequency histograms of the maximal deviations from the mean acceleration and graphs are the pdf curves within the ranges of the experimental data and for the estimated shape and scale parameters of the continuous Gamma family of probability distributions. **(H,I)** Summary of the right (red) and left (blue) wrist Gamma pdf patterns obtained within the temperature values determined using the methods of Figure 2 according to the minimal noise-to-signal values within the temperature ranges from the sensor’s readings. Each number on the graph represents the day of the recording in the month of May.

Once the proper regimes of noise-to-signal levels are determined from the motion-temperature data, and their rates of change obtained, we go back to the stochastic analyses of the acceleration data. The prior methods allow us to zoom in the month of highest (or lowest) change in activity across the longitudinal data. Figure 8 shows the frequency histograms of the right and left wrists data involving the maximal deviations from the mean acceleration obtained within the proper temperature intervals (those identified with the lowest noise-to-signal levels). The figure focuses on the month of May which Figure 7 identified as critical for the dominant hand. Notice the changes in the shape and width of these frequency histograms across the various sessions in May.

**Figure 9:**
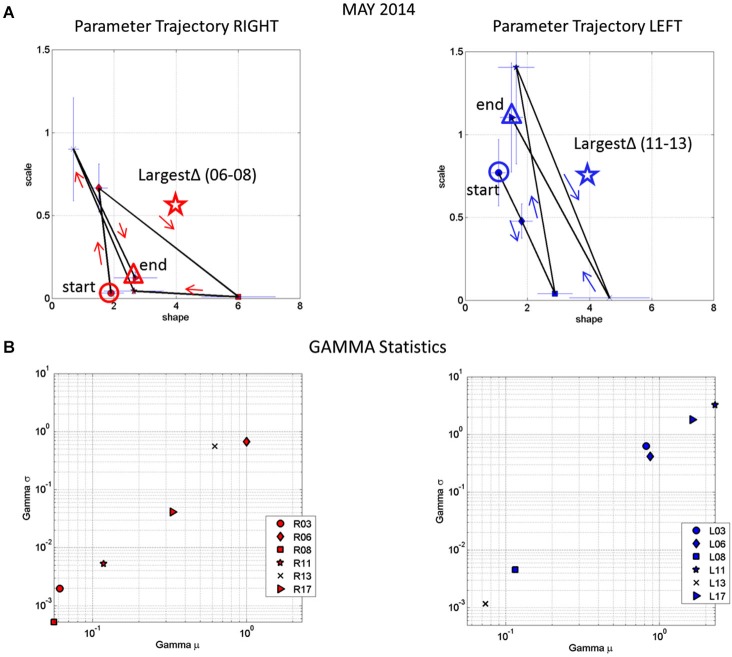
**Longitudinal trends in changes of stochastic signatures of acceleration variability (May). (A)** Trajectory across recording sessions of the estimated scale and shape parameters for maximal acceleration using the continuous Gamma family of probability distributions. Each point is plotted on the Gamma parameter plane with 95% confidence intervals. Arrows indicate the flow of the trajectory in the order in which the data were acquired from the start circle (May 06) to the end triangle (May 17th). The star is the point of maximal change in shape (towards symmetric Gaussian-like range of the Gamma plane) and drop in scale value (decrease in the noise-to-signal ratio). **(B)** Estimated mean and variance parameters of the Gamma probability distribution for each session (see legend with dates for right R and left L cases) with symbols corresponding as well to parameters in **(A)**. The log-log plot is used for better visualization. Notice that the values of the variance and the mean corresponding to the maximal shifts in stochastic parameters marked by stars in **(A)** are at the extreme locations of the Gamma-statistics plane.

Figure 9A tracks the stochastic trajectories of the estimated Gamma parameters for each wrist (corresponding to the acceleration-dependent motions) and identifies (with a star) in each case the session with the largest rate of change towards the regimes of lowest variability (most reliable) and most symmetric shape, towards systematic motions, away from the (most random) Exponential distribution regimes of the Gamma plane. The starting and ending points of the trajectories are also highlighted. Figure 9B shows for each day the Gamma estimated statistics (mean and variance) highlighting in the legend the dates of the sessions and the largest change in statistical regimes. Other analyzes of the rates of change in these estimated parameters were performed for the month of May and for other months as well. We report the results in the next section of the paper.

## Results

Identification of the motion regimes with the lowest noise-to-signal ratio per session enabled us to focus on the physiologically relevant motion data and examine the rates of change of the width and the shape of the frequency distributions of the maximal deviation from the mean acceleration. Given that there are many spontaneous motions in the patient, the purpose of these analyses was to discriminate random from systematic changes in shape and scale parameters, as well as to establish possible relations between motion and temperature data indicative of emerging volition in the movements.

**Figure 10:**
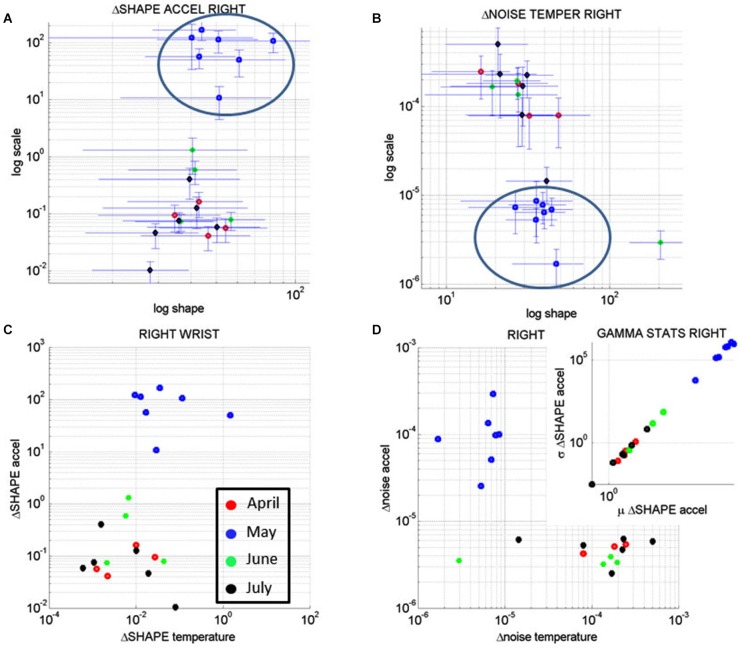
**Longitudinal analyses of the rates of change of the noise-to-signal levels of the acceleration as a function of the temperature readings. (A)** The changes in the shape of the distributions of the noise-to-signal values of the motion (maximal deviations from the mean acceleration) followed a Gamma distribution. They are plotted on the Gamma parameter plane. Notice that the stochastic signatures corresponding to the sessions recorded in May stand out from those in the other months. **(B)** Similar analyses in the rate of change of the noise-to-signal of the temperature of the right wrist single out the month of May as a separate cluster from all other readings. Lower changes in the noise level and overall higher values of the parameter indicating the shape of the distribution were registered in May, as compared to the other months. **(C)** The rates of change in the shape of the distributions characterizing the noise-to-signal levels of the acceleration as a function of the temperature were systematic during the month of May and clustered apart from the readings of the other months. **(D)** The stochastic signatures of the rates of change in the noise-to-signal levels of the acceleration expressed as a function of those of the temperature also clustered apart in May from the rest of the recordings in other months. Inset shows the estimated Gamma statistics for the rate of change in the shape of the Gamma distribution corresponding to the acceleration parameters with systematic increases in the variability of the maximal deviation from the average acceleration with increases in the mean value of this parameter.

Figure 10A shows the result of the analyses corresponding to the stochastic changes in the shape of the distribution estimated for each of the sessions of each month where the noise-to-signal was at its minimum. The frequency distribution of the rate of change of the shape parameter in each session was well fit by the Gamma family. The estimated shape and scale parameters are plotted with 95% confidence intervals on the (log-log) Gamma Plane. This plane shows a clear separation in the clustering of the points corresponding to the sessions in the month of May for the right wrist. This separation is consistent with the overall behavior of the changes in temperature and motion data identified in Figures 7C,D. The upward shift in this cluster along the vertical axis indicates an increase in the variability (the width) of the shapes of the distributions of the acceleration-dependent motion parameter. The rightwards shift of this cluster along the horizontal axis indicates systematic changes towards more symmetric shapes. More symmetric shapes indicate Gaussian-like behavior with a build up in the expected value of the parameters while shifts away from these regimes (towards the left of the shape axis) mark increase in randomness and total lack of volition.

In summary these analyses revealed that the rate of change of the shape parameter estimated from the linear accelerations with the lowest noise-to-signal values singled out May as the critical month. This was the month with highest variability in the change of the shape parameter of the maximal deviations from the mean acceleration, but it was also the month when these changes were the most systematic, predictive of a reliable expected value. In other words, the variability in the acceleration-dependent motion of the dominant hand was not random during the month of May. These motions were not spontaneous in nature as those with random patterns are. The rate of change in the stochastic patterns was highly systematic, as quantified by the shifts in the shape of the probability distribution of the acceleration dependent parameters.

Figure 10B shows the results from similar analyses as in Figure 10A but this time corresponding to the rate of change of the noise-to-signal levels in the surface skin temperature. The frequency distributions of the rate of change of surface skin temperature noise followed the Gamma distribution as well. We estimated the shape and scale parameters of each session with minimal acceleration-dependent motion noise and plotted the point from each session on the (log-log) Gamma plane with 95% confidence interval. The month of May once again stood out as a separate cluster with systematic shifts downwards towards regimes of reliable measurements (low noise) and shifts rightwards towards more systematic regimes tending towards symmetric (Gaussian) shapes of the distribution of the rate of change in temperature noise. These patterns were not present in the values registered by the left wrist. In the left wrist the points from the sessions in the month of May did not cluster apart from those estimated from the measurements taken in the other months. Unlike in the right wrist, no reliable and systematic changes were revealed in the motions of the left wrist during the month of May.

Figure 10C shows the patterns corresponding to the rate of change in the shape parameter discussed in Figures 10A,B for the acceleration-dependent motion as a function of the temperature. The points representing the month of May cluster apart from the rest. Here, in relation to the other months, May had larger values for the change in the shape of the acceleration-dependent distribution corresponding to larger values in the change of the shape of the temperature-dependent distribution. This indicates a systematic change in the shapes of these distributions towards more symmetric shapes: as the changes in the shape of the distributions of temperature became more systematic, so did the changes in the shape of the distributions of the maximal deviations from the mean acceleration. This means that in May the changes in the motions of the right wrist as a function of surface skin temperature were not random.

While Figure 10C speaks of systematic changes in the shapes of the parameters’ distributions, Figure 10D speaks of the changes in their noise-to-signal levels. There we see that in relation to other months, the measurements in the month of May stood out with lower changes in temperature noise and higher changes in acceleration noise. The rates of change in noise levels in temperature were steady, while the rates of change in the acceleration noise increased. There was more variability in the motion for steady temperature ranges. Yet this variability was systematic according to the statistics of the shape values of the distribution of motion parameters shown in the inset.

The inset zooms in the Gamma statistics of the changes in the shape of the distributions of maximal deviation from the mean acceleration. The figure shows that in May the motions were more systematic than in the other months and their variability in the shape of the distribution was higher. In particular, by May 17th the changes in surface skin temperature were steadier as the changes in motion patterns turned more systematic (as revealed by the higher values of the shape of the distribution of the relevant acceleration and temperature dependent parameters).

Patient AB had the C-section delivery of her baby boy on May 22, 2014. All the data preceding that date indicated patterns of systematic variability in her motions from the dominant (right) hand that were absent in the motions from her non-dominant (left) hand. Furthermore, the medical records indicated the formation of a blood clot in the right arm after May. Figure 7D shows a slow gradual increase in the changes in surface skin temperature for the left wrist that also coincided with higher levels of motion. These motions from the left wrist however had no discernable patterns of systematic changes in variability levels as those observed in May. The motions registered in the left wrist were truly spontaneous in nature (random) whereas those of the right wrist were reliably predictable of an expected value.

## Discussion

This paper introduces new methods to assess in a personalized manner the day-by-day longitudinal progression of body motions as a function of surface skin temperature using wearable sensors. The statistical metrics introduced here may permit the continuous longitudinal assessment of patients as they move and as they undergo changes in physiological states. We have used a particular case of a patient with severe brain trauma to illustrate the methods. Yet these methods can be generally extended and used in other patients as well. These methods do not assume population statistics or expected values of the parameters of interest. Instead, they empirically estimate the probability distributions most likely underlying the changes in motion and physiologically relevant parameters registered in tandem within each daily session and longitudinally over months. The methods focus on the rates of change of these parameters’ statistics along a continuum.

A surprising revelation from these analyses was that not all motions recorded by wearable sensors were physiologically relevant. We found a great deal of instrumentation noise that we had to separate from the signal in order to perform appropriate analyses on the motion data. This is important in light of the general use of wearable sensors in the market to track activity, wellness and fitness. Here we found high levels of noise-to-signal ratio in the acceleration data under unrealistic regimes of skin surface temperature. We were able to further use the surface skin temperature as a natural filter to help us separate the random rates of change of the noise levels in the motion from systematic rates of change. We also distinguished systematic from random changes in the shape of the distributions of these parameters.

These data analyses suggest that in general more motion registered by accelerometers does not imply that there is more neural control of movements. The registration of higher acceleration values should not be associated with more volitional control or intent in the motions. Instead, one should separate the noisy data and assess the levels of reliable and systematic changes in the motion data with low noise-to-signal ratio. In these sensors a layer of noise, particularly at low levels of temperature rendered irrelevant a large portion of high levels of motions registered by the tri-axial linear accelerometers. The lower temperature regimes coincided with motion data that was predominantly noisy. This was consistently the case across all sessions of recordings.

We note here that Newtonian mechanics concerned with acceleration estimations has no known relation to thermodynamics. The laws of mechanics governing physical motions were derived for inanimate objects and rigid bodies, rather than for biological bodies in motion undergoing physiological changes that impact the motions’ variability. Such changes are guided by feedback from sensory nerves conducting information from the peripheral to the central nervous system about pain, temperature and motion/touch/pressure. Although the field of neural control of movement employs primarily Newtonian mechanics in the analyses and modelings of behavioral states (Shadmehr and Wise, 2005), it may be important to introduce new ways of examining motion data in tandem with physiologically relevant measurements (such as temperature, heartbeat, breathing patterns, etc.) of use in clinical settings. An approach such as the one introduced here would then enable us to better understand the nature of motion data that is also governed by a nervous system under volitional control, rather than exclusively described by the physical laws of motion. The motor output variability registered in tandem with the surface skin temperature helped us unambiguously distinguish random (spontaneous) patterns from systematic, reorganized (predictable) patterns. We may have characterized a degree of volition in the dominant wrist as the patient repeatedly touched her abdomen during the contractions preceding the day of the C-section.

While analyses and modeling of motion data is the exclusive focus of the field of neural control of movements without regards of physiological data, the medical field follows a complementary approach to patient assessment. In the clinical settings, measurements of physiological data such as temperature, breathing, heartbeat, blood pressure, etc. are routinely taken from the patient. These measurements are taken in isolation, without considering possible relationships to bodily motion patterns.

The human body is in constant motion in tandem with other physiological patterns of the person. Such patterns fluctuate and change over time. In clinical settings the absolute values of the parameters of interest are often registered, but very little is said about the trends and fluctuations of their rates of change over time. Here we have shown that the rates of change of those parameters over time contained information predictive of a relevant upcoming event. In particular we were able to blindly identify May as the month of highest relevance in these longitudinal data sets. A dramatic and sharp change in the patterns of motion and surface skin temperature of this patient’s dominant hand manifested in May preceding the birth of her baby boy by C-section.

These metrics may be of use to monitor critical events during pregnancy and foretell (and possibly prevent) potential problems. It is possible that we may have even captured and characterized here in this patient the patterns of noise-to-signal corresponding to the painful contractions that are known to precede birth, as her hand moved to her abdominal area. It will be interesting to repeat this study systematically in a large number of pregnant women of different ages. We would be able to characterize with unprecedented precision the risk of miscarriage as a function of age, as well as various individualized physiological scales of painful contractions as a function of temperature and motion profiles, among other symptoms during pregnancy.

The critical task of characterizing longitudinally the individualized profiles of various physiological stages of pregnancy in an objective, non-invasive manner would be highly feasible now using our new analytics in tandem with a broad range of wearable sensors available in the market. The current market offers sensors that capture heart rate variability, electro dermal activities, and blood-volume levels, among others. The various outcomes of these biomarkers are currently examined in isolation during visits to the clinic. These new analytics offer the possibility of integrating the physiological signals with the motion’s temporal profiles to provide a multi-dimensional profile tractable longitudinally at home and during the visits to the clinic. Pregnant patients increase their visits to the clinicians during the last trimester and other techniques are used to monitor their progress (Reece and Hobbins, 2007). It would be ideal to team up with an antenatal and perinatal expert to be able to compare and combine the methods presented here with currently used neuroimaging techniques, ultrasound among others.

We also suggest that in the future by combining the motion and the physiological measures (registered in tandem) we could better and continuously monitor patients with post trauma to the brain (independent of the type of trauma). We could better understand the course of individual changes in their motions and body physiology as the patient receives therapies and as the patient undergoes drug treatments. We could find new ways to objectively track the progress of patients as they recover from brain trauma, identify critical points along the evolution of the person and assess the effectiveness of treatments in non-invasive ways. We could do all of this continuously at home or in the clinic by simply using off-the-shelf wearable sensing technology broadly available today.

In summary these sensor’s physiological data were able to blindly forecast what without a doubt would be considered the most important day in a woman’s biological lifecycle. They did so even under a coma state. The information revealed by this new analytical technology could be potentially of use to individually track the longitudinal patterns of other patients with post trauma to the brain and tailor their treatments accordingly. These new metrics may bring us a step closer towards true personalized medical practices.

## Conflict of Interest Statement

The authors declare that the research was conducted in the absence of any commercial or financial relationships that could be construed as a potential conflict of interest.
